# Network Analysis of Differential Expression for the Identification of Disease-Causing Genes

**DOI:** 10.1371/journal.pone.0005526

**Published:** 2009-05-13

**Authors:** Daniela Nitsch, Léon-Charles Tranchevent, Bernard Thienpont, Lieven Thorrez, Hilde Van Esch, Koenraad Devriendt, Yves Moreau

**Affiliations:** 1 Department of Electrical Engineering (ESAT-SCD) Katholieke Universiteit Leuven, Leuven, Belgium; 2 Center for Human Genetics, University Hospitals Leuven, Leuven, Belgium; Universidade de Sao Paulo, Brazil

## Abstract

Genetic studies (in particular linkage and association studies) identify chromosomal regions involved in a disease or phenotype of interest, but those regions often contain many candidate genes, only a few of which can be followed-up for biological validation. Recently, computational methods to identify (prioritize) the most promising candidates within a region have been proposed, but they are usually not applicable to cases where little is known about the phenotype (no or few confirmed disease genes, fragmentary understanding of the biological cascades involved). We seek to overcome this limitation by replacing knowledge about the biological process by experimental data on differential gene expression between affected and healthy individuals. Considering the problem from the perspective of a gene/protein network, we assess a candidate gene by considering the level of differential expression in its *neighborhood* under the assumption that strong candidates will tend to be surrounded by differentially expressed neighbors. We define a notion of *soft* neighborhood where each gene is given a contributing weight, which decreases with the distance from the candidate gene on the protein network. To account for multiple paths between genes, we define the distance using the Laplacian exponential diffusion kernel. We score candidates by aggregating the differential expression of neighbors weighted as a function of distance. Through a randomization procedure, we rank candidates by *p*-values. We illustrate our approach on four monogenic diseases and successfully prioritize the known disease causing genes.

## Introduction

Genetic studies, including cytogenetic, linkage, and association studies, can identify chromosomal regions associated with a disease or phenotype of interest. Similarly, high-throughput ‘omics’ experiments identify genes or proteins implicated in a biological process of interest. In both cases, biologists are often confronted with long lists of tens or hundreds of candidate genes among which they need to select a limited number of candidates for further validation. This problem has been termed *gene prioritization* [1]. Recently, computational methods for prioritizing candidate genes have been proposed. They usually rank candidates by matching their information across multiple data sources against a profile derived from a set of genes, pathways, or biological processes already known to be involved in the phenotype. A frequent objection to this class of methods is that they cannot be applied to cases where little is known about the molecular basis of the phenotype (no confirmed disease genes, fragmentary understanding of the biological cascades involved). We seek to overcome this limitation by replacing knowledge about the biological process by experimental data on differential gene expression between affected and healthy individuals. Considering the problem from the perspective of a gene/protein network, we assess a candidate gene by considering the level of differential expression in its *neighborhood* under the assumption that strong candidates will tend to be surrounded by differentially expressed neighbors.

A number of methods are currently available for gene prioritization. *Aerts et al. (2006)* developed Endeavour, a gene prioritization method that ranks candidate genes based on their similarity to genes already involved in the biological process of interest, using multiple data sources (e.g., sequence, expression, literature) [2]. Similarly, *Köhler et al. (2008)* developed GeneWanderer, a method for prioritizing candidate genes by the use of the random walk analysis that defined similarities in protein-protein interactions (PPI) networks [3]. Their global distance measure defines the similarity between genes within the global network and ranks candidate genes on the basis of their similarity to known disease genes. They hypothesized that a global network-similarity measure captures associations between disease proteins better than algorithms based on direct interactions or shortest paths between disease genes. *Franke et al. (2006)* also incorporated the interactions between genes in a network to prioritize candidate genes [4]. They developed a human gene network that integrates information on genes and their functions. Their method, Prioritizer, ranks candidate genes on the basis of their interactions. They analyze susceptibility loci and investigate whether genes from different loci can be linked to each other. *Lage et al. (2007)* developed a phenome-interactome network that integrates phenotypic literature information from OMIM with a cross-species PPI network [5]. They implemented a Bayesian disease gene predictor that computes for each candidate gene the probability that it is the disease-related gene. High probabilities are assigned to genes that interact with genes that are already associated with phenotypically-related disorders. *Chuang et al. (2007)* developed a network analysis method by applying a protein network-based approach that identifies biomarkers not as individual genes but as subnetworks extracted from protein interaction databases [6]. To find associations between phenotypes and subnetworks, they developed a scoring based on mutual information measure. Although their methodology resembles that of gene prioritization, their method is related to biomarker discovery rather than prioritization.

Most of available prioritization methods have in common that they require knowledge about the disease to identify putative disease genes, for example in the form of a set of genes, pathways, or gene ontology categories known to be implicated in the disease. They then rank candidate genes through “guilt by association” methods across a variety of data sources. But when nothing or only little is known about a disease, these methods will be inapplicable or ineffective. While it is extremely useful to incrementally add disease genes to phenotypes whose molecular basis is reasonably well characterized, there is strong demand from geneticists for methods that could help in the more difficult case of disorders for which the molecular basis is not yet elucidated. Currently, there are not yet truly effective prioritization methods for this case.

We seek to overcome this limitation by replacing knowledge about the biological process by experimental data on differential gene expression between affected and healthy individuals. Our method is a generalization, from a systems biology perspective, of a standard procedure for assessing candidate genes in genetic studies. A standard genetic procedure to analyze candidate genes is to check the expression level of a candidate gene in patient-derived material against wild type (typically in fibroblast or immortalized lymphoblastoid cell lines). Candidates for which a significant difference is observed between the two groups are considered promising. However, for many genetic diseases (such as diseases arising from point mutations in coding regions), there is no guarantee that the expression level of the disease gene itself is affected (although this is possible through feedback effects). Rather, genes “downstream” of the disease gene are those whose expression will be affected. Instead of considering genes in isolation, we consider the differential expression data now at the level of a gene/protein network. If we look at expression patterns mapped on a gene network, we therefore expect that we will observe a disrupted expression module around the disease gene. Other candidate genes, which are not causally related to the phenotype, should not be part of such a disrupted expression module. For this reason, the entire affected neighborhood has to be considered for each candidate gene instead of only taking its own expression level into account.

When considering the expression data at the network level, we need to rely on a gene/protein network. Originally, protein-protein interaction networks from data on putative physical interaction between proteins. More recently, protein networks combining a variety of information sources have been proposed. A link in such a network does not necessarily imply physical interaction between two proteins, but rather some form of association resulting from different types of data (actual interaction, membership in the same pathway, coexpression, literature cooccurrence, etc.) We will call such a network, a protein association network, or protein network for short. Note that in such networks no distinction is made between gene and protein, or multiple isoforms. Furthermore, protein networks are far from complete, and dealing with direct protein-protein interactions may be suboptimal since protein networks are still sparse because of many unknown components and pathways [4], [7]. Also, procedures that define neighborhoods in terms of the minimum number of steps from a given gene suffer from the “small world” effect (i.e., the number of neighbors of a given gene grows rapidly with the number of steps taken along the network). To overcome those potential limitations, we chose to use a global distance network that considers both direct and indirect paths in the network [3]. By considering indirect interactions in a protein network, missing links in the network can be compensated. Thus, a global distance network is more densely connected than the sparse protein network from which it is derived. Specifically, our method derives the global distance network from a functional protein association network (STRING [8]) using kernel methods. STRING integrates both protein-protein interactions as well as predicted interactions based on comparative genomics and text mining [8].

**Figure 1:**
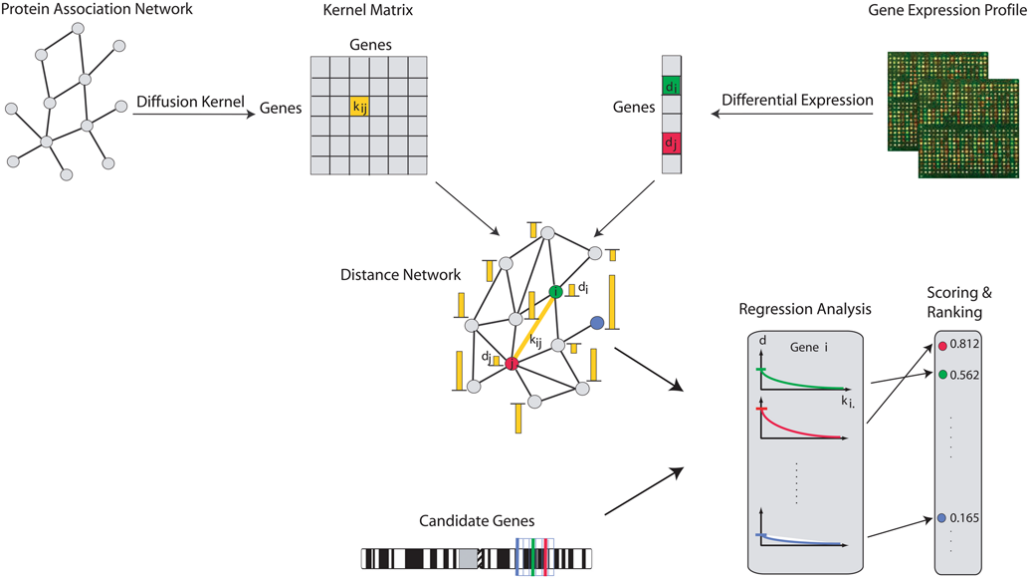
Overview of the method. (1) From a protein association network a global distance network is computed based on a kernel method (e.g., the Laplacian exponential diffusion kernel). (2) A disease related microarray experiment is required from which the differential expression levels of all genes in the network are determined. (3) The differential expression levels of the genes are mapped on the global distance network. (4) A set of candidate genes is required (e.g., from a linkage study). (5) For each candidate gene its differentially expressed neighborhood is identified by a regression analysis. (6) Based on the regression analysis each candidate gene receives a score. (7) The candidate genes are ranked by their scores.

Figure 1 shows an overview of our approach. For each gene in the network, its differential expression level is determined by transcriptome-wide microarray experiments of mutant vs. wild-type lines. Then differentially expressed neighborhoods in this network are considered for all candidate genes from a chromosomal region of interest (e.g., identified in a linkage study). Candidate genes with genes in their neighborhood having highly differentially expressed levels are strong candidates. The neighborhood of a candidate gene is defined by direct connections in the global distance network, whereby its size can vary. The smaller the distance from a neighboring gene to the candidate, the closer the neighboring gene is in the network. We have chosen to work with the notion of a *soft neighborhood* by which we mean that the neighborhood of a gene is not a limited set of gene, but rather a weighting function that decreases monotonically as a function of the distance from the gene, but potentially covers the whole network (this idea is reminiscent of the idea of fuzzy membership in fuzzy c-means clustering).

To identify candidates belonging to a significantly disrupted expression module, we have developed a novel randomization method that identifies modules with significantly affected genes. Each candidate receives a score based on the analysis of the differential expression along its neighborhood. The level of differential expression of each gene is weighted by its network distance from the candidate and summed up over all genes. Therefore, the higher the differential expression levels of neighboring genes with small distances are, the higher the score. To determine a candidate gene's significance, a *p*-value based on a randomization procedure is computed. If a candidate belongs to a significant disrupted expression module in the network, its *p*-value is expected to be significant.

Evaluating such a method *in silico* is obviously challenging because if we make predictions on actual diseases where no genes are known, the only way to validate those predictions will be to carry out a full biological validation. Moreover, the kind of expression data needed for our analysis will be currently available only for very few diseases because at this point biologists mostly carry out this type of experiments for diseases for which the cause is known. In a first step, we therefore mimic the situation by taking known disease genes for which expression data for patients versus controls is available and attempt to recover the gene mutated in those patients. We present four distinct data sets for which we could successfully prioritize the disease-causing gene for each data set. The average rank of the known disease genes was approx. 4 out of approx. 120 candidate genes (see Results). Thus, our method can detect unknown disease causing genes by identifying the most disrupted expression modules in the global distance network. In a second step, using one of the only available human expression data sets for a disorder of unclear etiology, we have applied our method to the polygenic disorder Stein-Levental syndrome for which no disease gene is known. We could confirm the influence of two candidate genes: fibrillin 3 (FBN3, for which a susceptibility allele has been identified) and follistatin (FST, for which association with a Stein-Levental syndrome related metabolic phenotype has been shown). Finally, we suggest a new candidate gene (DEAD box 4) potentially involved in this disease.

## Results

Our distance network was derived from the STRING database [8], from which we used all data types provided (genomic context, high-throughput experiments, coexpression data, and previous knowledge). The resulting network detects all direct and indirect connections of genes and represents a notion of global distance measure (see Materials and Methods).

We illustrate our method by its application to the analysis of constitutional genetic disorders caused by a single gene mutation. Following the current practice of assessing candidate gene expression in EBV cell lines or fibroblast cultures in mutant against wild type, we consider expression data from such biological material (or other accessible tissue biopsies). There is however only a limited number of such data sets publicly available through the ArrayExpress [9] and Gene Expression Omnibus [10] repositories. We present here the results of the method on four case studies.

We distributed all signals randomly over the network to estimate the significance of the candidates. In an adequate data set, at least one gene should be found with a significant p-value (i.e., *p*<0.05). We then assessed how high the actual disease gene ranked and whether its score was significant.

### Case studies

We have evaluated our approach on four data sets: (1) fragile X syndrome (FXS) [11] caused by mutation of FMR1 (fragile X mental retardation 1), (2) Marfan syndrome (MFS) [12] caused by mutation of FBN1 (fibrillin 1), (3) cystic fibrosis (CF) [13] caused by mutation of CFTR, and (4) Becker muscular dystrophy (BMD) [14] caused by mutation of DMD.

For each data set, we have determined a set of candidate genes by taking the genes within a set of chromosomal bands centered on the disease-causing or disease-related gene to gather approx. 120 genes. Genes that were absent in our distance network were not further considered. For finding differentially expressed genes in the network we computed the fold-change for each gene in the genome (see Materials and Methods). Beside the scores of the candidates and the fold-change derived from the microarray experiments, we also present known links to similar diseases with related phenotypes.

Each data set and the high-ranking candidates (i.e., those that have a significant p-value) that are phenotypically linked to related diseases [15]–[21] are characterized in the Supplementary Materials S1. This demonstrates the significance of the method and shows that not only the actual disease-causing gene can be identified, but also related genes that may also be involved in this disease.

### Neighborhood

Determining an adequate size for the neighborhood that influences the score of a candidate is a challenge and influences the ranking. For three data sets (fragile X syndrome, Marfan syndrome and Cystic fibrosis) we have determined small neighborhoods of 150 or 20 neighboring genes, because for these data sets we obtained the best signal for small neighborhoods (data not shown) after applying the Fisher omnibus statistics (see Materials and Methods for more details). However, for one data set (Becker muscular dystrophy) we have determined a larger neighborhood of 2000 neighboring genes due to high signal for a large neighborhood (data not shown).

To illustrate the difference between disrupted expression neighborhoods of significant candidate genes and not significant genes, we have added graphs containing the queried neighborhood of the candidates (Figures S1, S2, S3). These graphs show the differential expression levels of the neighbors and their distances to the candidate gene. We can observe that the closest neighborhood of a significant candidate is highly differentially expressed and belongs to a more disrupted expression module than the neighborhood of a nonsignificant candidate.

### Significance

To determine a candidate gene's significance, the differential expression levels are distributed randomly over the network. The candidate's score is compared with the distribution of the randomly generated scores that leads to a *p*-value. If a candidate's score is larger than 95% of all randomized scores, this candidate gene can be considered as belonging to a significant disrupted expression module in the network.

**Figure 2:**
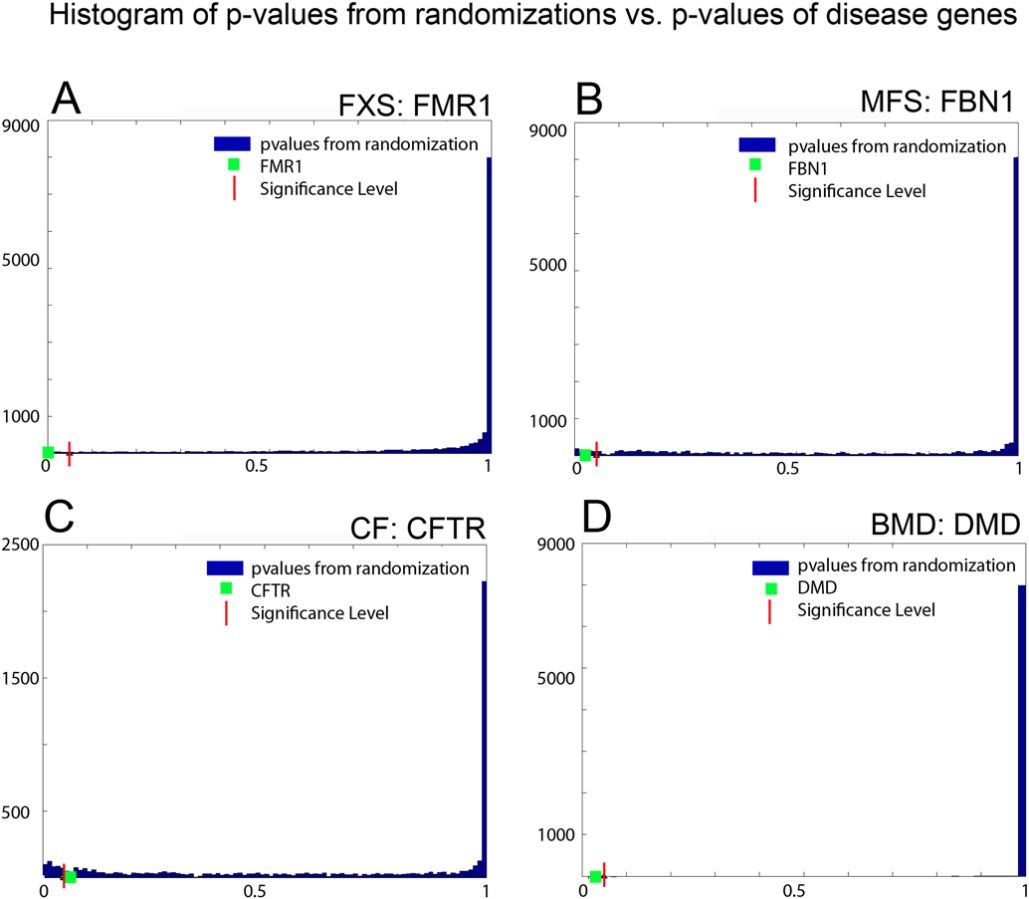
Histogram of *p*-values from randomizations vs. *p*-values of the disease genes FMR1, FBN1, CFTR, and DMD. This figure displays the histogram of *p*-values obtained after 10,000 randomizations, and the *p*-values of the disease-causing genes from all four benchmark data sets. It can be observed that in all data sets only a small set of genes have significant *p*-values (i.e., they belong to disrupted expression modules) and all disease causing genes are among them.

Figure 2 shows for all data sets the distribution of *p*-values after 10,000 randomizations, and the *p*-values of the disease genes whereby all were assigned significant *p*-values. These plots demonstrate that a clear distinction could be made between significant genes with low *p*-values and all other genes, and that the disease genes could be identified by their significant *p*-values and their high ranking.

### Ranking

Tables S1, S2, S3, S4 show the results of the ranking of the evaluation data sets. In all four benchmark data sets, the disease causing genes were ranked in the top 10 (FXS: 1^st^ position, MFS: 5^th^ position, CF: 7^th^ position, BMD: 2^nd^ position) out of lists containing approximately 120 candidate genes (see Supplementary Material), and all were assigned significant *p*-values. For all data sets, we could identify several disrupted expression modules of different sizes around a candidate gene. For two data sets (FXS and CF), some of the top ranked genes were already known to be directly associated with a related disease or phenotype [15]–[21] that emphasizes the significance of this result. We could not only successfully identify the disease genes, but also genes that correlate with the corresponding phenotype.

One of our aims was to develop a method that is independent of the differential expression levels of a candidate gene itself. Therefore, for evaluation purposes, we did *not* take the differential expression levels of the candidates into account but, preferably, the levels of their neighboring genes. Although in practice, we should obviously take into account the differential expression level of the candidate itself (because the disease gene can be disrupted through feedback effects). Our rankings in Tables S1, S2, S3, S4 demonstrate clearly that the ranking orders do not depend on the up- or down-regulation of the candidate genes themselves, but rather on the effects of being positioned in their neighborhoods. Thus, genes that are not differentially expressed can rank higher than highly differentially expressed genes as long as their neighborhood is differentially expressed.

### Application to Stein-Levental syndrome

We have applied our approach to the Stein-Levental syndrome [22], which is characterized by obesity, hyperandrogenism, and chronic anovulation in women. Stein-Levental syndrome is an endocrine disorder that affects approximately 5% of women and is a leading cause of infertility. This syndrome is believed to be oligogenic (i.e., caused by the effect of a limited number of genes) rather than monogenic [23]. Follistatin (FST) was originally proposed as a candidate for Stein-Levental syndrome by linkage and association studies [24], but is now thought to be rather associated with key androgenic phenotypes of Stein-Levental syndrome but not with the disease itself. These results suggest the existence of another disease-causing gene for Stein-Levental syndrome in the vicinity of FST (rather than FST itself). Recently, a Stein-Levental syndrome susceptibility locus was identified at 19p13.2 [25] and further association studies have suggested fibrillin 3 (FBN3) as the Stein-Levental syndrome susceptibility locus [26]. However, beside FST and FBN3 other candidate genes have been studied, often with inconsistent results [27].

We have determined two sets of candidate genes located on the chromosomal region of FST (chr5q11.2) and FBN3 (chr19p13.2). Both genes (FST, FBN3) were ranked high by our method (Tables S5, S6) and we could confirm their important role in this disease. We further detected the DEAD box 4 gene that is located on chr5q11.2 and that was ranked on the top position with a significant *p*-value (Table S5). We suggest that DEAD box 4 is a new candidate gene for Stein-Levental syndrome because of association with stem cell recruitment to the ovaries, interaction with the microRNA processing machinery, and impact on apoptosis [28]–[35].

## Discussion

As mentioned in the introduction, there are several known methods to prioritize candidate genes. These approaches can be split into methods that need known disease-gene associations about the disease [2], [3], [5] and methods without this precondition [4]. If little knowledge is available for a specific disease, the methods that require known disease genes will be ineffective. *Franke et al. (2006)* presented a method to prioritize candidate genes without a training set [4], but without using expression data to evaluate the candidate genes. Among a set of disease loci, they will look for pairs, triples, etc. of genes at different loci for which interaction has been described. Such sets of interacting genes are considered more likely candidates for causing the disease at the different loci. Because of the combinatorial nature of the test, the method does not have high statistical power. Along other lines, *Chuang et al. (2007)* introduced a network analysis method using expression data [6]. However, they focused on finding active subnetworks and biomarkers in cancer and developing a methodology that is not directly related to our problem. Our strategy differs as we focused on searching for disease genes for which genetic mutation causes constitutional disorders.

The strength and uniqueness of our approach is that we substitute disease-specific experimental data (in our case expression data) for the prior knowledge of the molecular basis of the disorder. ENDEAVOUR [2] could also incorporate disease-specific expression data but did not use any network analysis concepts and would still mostly rely on the knowledge from the set of known genes for the disease. Among all other methods, only *Chuang et al. (2007)* leverages experimental data [6], however with an entirely different scope and method. Essentially, although existing methods could be applied on the known benchmark cases, none of them would be directly applicable to the actual situation where we want to use our method, which is when the molecular basis of the disease is unknown (with the case of Stein-Levental syndrome as an illustration).

A first question is in which setups our method is applicable. We have chosen here to apply our method in a setup where a locus is known for the disease. Although this is not a strict requirement and genes can be prioritized on a genomewide basis, it has the advantage of limiting the number of candidates tested and therefore limiting the number of false positives. It also guarantees that at least one gene must be associated with the disease. However, among genetic studies, while the method is relevant to linkage and association studies, it may not be applicable to loci detected by cytogenetic studies of patients with genomic deletions or duplications. Indeed, in this case, multiple genes are affected by a copy number change, so that the expression data can be expected to be the superposition of the downstream effects of all the affected genes (although it could be that most of the phenotype is explained by a single critical gene that dominates the downstream cascade of expression dysregulation, in which case the method may still be applicable). Although the concept of our method may seem at first tailored to monogenic disorders, it is more broadly applicable. This is demonstrated by our case study on the polygenic disorder Stein-Levental syndrome, where we could detect FBN3 and FST as related to the disease.

Several factors influence the performance of our prioritization method. First, the quality and coverage of the network around the actual disease gene will be a strict bottleneck. For example, an isolated gene with no edges in the network can never be effectively prioritized by our method (except by relying solely on its own expression data). We have chosen to use the STRING database (version 7.1) because it is built by taking into account multiple heterogeneous data sources. This results in a large network with a good coverage (human: 16,050 genes, mouse: 16,566 genes). Errors in the network (caused by incorrect gene annotation, unreliable functional annotation, etc.) may both cause false negatives (missing the disease gene) as false positives (genes incorrectly identified as promising because of incorrect association with other genes). Continuing improvements in the quality of protein association networks will contribute to increased effectiveness of the proposed method. Moreover, protein association networks are naïve in terms of alternative transcripts of genes and protein isoforms. At this moment, no distinction is possible among them. It is also unclear what the quality and coverage of nonprotein-coding genes is in current protein association networks – although our candidate gene DEAD box 4 for Stein-Levental syndrome suggests a possible role for the nonprotein-coding genes PIWIL2 (MILI) and DICER1. Several alternatives exist to the STRING network including BioGRID, IntAct, IntNetDb, HPRD, and Dip, and we will study the impact on performance of the choice of network in follow-up work.

Second, the quality and the relevance of the expression data will greatly influence the results of the method. Poorly collected samples (patients not actually sharing the same molecular phenotype) or disease heterogeneity (multiple genes or pathways leading to similar phenotypes) will obscure the expression pattern and will make picking up a meaningful signal more difficult. Similarly, lack of access to the most relevant cell types (biopsies cannot be performed arbitrarily on patients) can be a limiting factor for the method. If the relevant molecular cascades are simply not active in the cell type assessed, differences in expression may be meaningless. The idea is that the pattern of differential expression is as concentrated as possible on the network so that the affected subnetworks can be effectively identified. Some mutations may lead to extensive downstream cascades that may be reflected in network patterns that are too broad to effectively pinpoint the disease-causing gene. A promising experimental direction for focusing the expression patterns towards the disease-causing gene would be to use more sophisticated factorial disease for the microarray experiment. If we can identify a stimulus that is incorrectly processed in affected individuals (e.g., a metabolite or a protein), we could attempt to perform an expression profiling experiment where patient and control material receive a treatment that triggers the affected cascades. The differential expression response will tend be more tightly focused towards those genes that are essential for the difference in response to the treatment between affected vs. non-affected individuals.

Third, an important parameter of the method is the neighborhood size or, the scale parameter of our weighting function that defines it. To study the neighborhood of the candidate genes in order to identify disrupted expression modules in the distance network, we had to bound the neighborhood to a limited size because the network was very densely connected, and thus the neighborhood of a candidate gene consisted of almost all genes in the network. This limitation was done by only considering the neighboring genes with the smallest distances to the candidate (any gene further than a certain threshold was not considered). We wanted the size of the neighborhood to be dependent on the disruption we found in the network. We determined this size by analyzing the observed signals obtained by applying the Fisher omnibus statistic to the list of candidate genes for different neighborhood sizes, and choosing the size for which we caught the best signal as the most reliable one. For three data sets (FXS, MFS, CF), we determined a small neighborhood because we observed best signal for small neighborhoods (data not shown). However, for the fourth data set (BMD), we determined a larger neighborhood because we could not observe a strong signal for smaller neighborhoods (data not shown). Therefore, we had to expand the number of neighboring genes that were taken into account for finding highly differentially expressed neighborhoods. This difference showed us that the size of the neighborhood can differ and is dependent on the number of affected genes in the disease and their biological pathways.

If we chose a neighborhood size for which we caught a weak signal, the ranking could produce an unreliable result. For example, *β* = 0.5 for FXS [11] that leads to a neighborhood size of 20 genes (see Table S9) would capture no meaningful signal from the neighbors and would lead to an unreliable ranking. Instead, a ranking for which a strong signal regarding to the significance is observed can be seen as a reliable result. Many factors influence the selection of an optimal neighborhood size: the shape of the weighting window, the density of the network (i.e., average node degree), the choice of the index of differential expression (here, the logarithm of the fold change), and so on. Further optimization of all the parameters will be necessary to devise an optimal procedure for neighborhood size selection.

Fourth, technical details of the mathematical model could have a substantial influence on the performance. Our purpose was to identify all direct and indirect connections in the functional protein association network from STRING that leads to a densely connected (global distance) network. We computed the Laplacian Exponential Diffusion Kernel [36] to obtain a global distance network. Following *Fouss et al. (2006)*, there exists several kernels on graphs, such as the Exponential Diffusion Kernel, the regularized Laplacian Kernel, the von Neumann Diffusion Kernel, and the Commute Time Kernel [37]. We chose the Laplacian Exponential Diffusion Kernel because *Köhler et al. (2008)* already applied this kernel as a good performing kernel to construct a distance network [3]. Furthermore *Fouss et al. (2006)* observed that this kernel performs systematically better than their corresponding adjacency matrix-based kernels (Exponential Diffusion Kernel, von Neumann Diffusion Kernel) [37]. However, a more systematic assessment on the effect of the kernel and its comparison to shortest-path, direct-interaction-only, or other methods, such as GTOM [38] is certainly in order. To efficiently compute the kernel matrix we applied the Cholesky Decomposition (see Materials and Methods) because the naïve computation was too time consuming. However, by calculating the Cholesky Decomposition we could compute the kernel method successfully with full accuracy (see Tables S7, S8). As a measure for determining the change in expression of a gene we chose the fold-change between control and experiment [39]. It is a commonly used measure (e.g., [11]–[14]), but numerous alternatives exist and will be compared in future work, such as differential expression indices expressed in terms of *z*-scores or log *p*-values, or combining the differential expression level and its statistical significance.

There are certainly diseases for which the candidate genes show no significances (i.e., there are no significant p-values that distinguish significant from not significant genes). In this case the disease does not lead to detectable disrupted expression modules or affected pathways in the network. Although it would be preferable to detect the disease gene, the ability of the method to return a negative result is an asset of the method. Indeed the performance is greatly influenced by the quality of the expression data, the quality of the protein network around the actual disease gene, and the underlying biology (i.e., existence of actually disrupted expression modules) so that the method cannot be expected to work optimally on every disorder. Therefore, the ability to assess statistical significant and potentially return a negative result (i.e., “no strong candidate found”) makes it possible to avoid pursuing “best” candidates that are actually not promising. There are also cases for which our method does return significant genes among which the known disease causing gene can however not be found. For those cases, one or several disrupted expression modules are found but none of them is centered on the disease-causing gene. This can be explained by the fact that even a single-gene disorder can result in the disruption of several pathways (downstream of the original pathway), making therefore the signal we are looking for more difficult to detect. This reflects that the current method is not suitable for all single gene disorders.

We applied our approach to Stein-Levental syndrome [22] and could identify two important candidate genes (FST and FBN3) from two different chromosomal regions [23]–[26]. We further detected the DEAD box 4 gene that is located on chr5q11.2 and that was ranked on the top position with a significant p-value. DEAD box polypeptide 4 (synonyms Mvh and Vasa) is an RNA helicase and is used commonly as a marker for germline cells [28], [29]. Although little is know about the molecular function of DEAD box 4 in mammals, there are several reasons why Dead box 4 is a plausible candidate gene for Stein-Levental syndrome. First, in male mice loss of Dead box 4 results in infertility due to suspension of premeiotic differentiation of spermatogenic cells [30]. Female knockout mice do not show any obvious reproductive defects, but it is plausible that the effect may be less dramatic in females, leading only to a (partial) arrest in follicle development. One of the genes that fail to be expressed in a mouse Dead box 4 knockout is Aven, a caspase inhibitor [29]. Failure to express Aven thus may result in altered apoptosis control. Stein-Levental syndrome is characterized by follicular arrest, where several follicles develop to a size of 5–7 mm but not further. Since primary follicles secrete androgens, too many arrested follicles likely lead to elevated androgen levels, a hallmark of Stein-Levental syndrome. Second, DEAD box 4 interacts with Dicer1 and is colocalized with MILI [31]. Dicer1 processes miRNA precursors to mature miRNAs which are incorporated in the RISC complex. These miRNA-RISC complexes then exert a broad posttranscriptional control on many mRNAs. Mili belongs to the Piwi family, encodes a component of the RISC complex and is expressed at early stages of oocyte growth [32] and crucial for progression through spermatogenesis [33]. In Drosophila, the loss of Piwi function leads to the failure of germline cyst formation [34]. As a third potential mechanism, altered DEAD box 4 may influence stem cell recruitment to the ovaries. *Johnson et al.* have demonstrated that stem cells from bone marrow and peripheral blood which are also marked with the presence of DEAD box 4, can migrate to the ovaries of sterilized females and give rise to oocyte-containing follicles. Interestingly, the level of DEAD box 4 is influenced by the estrous cycle [35].

In summary, we have developed a novel gene prioritization approach that substitutes expression data to prior knowledge of the molecular basis of the disease, as required by existing methods. Our method ranks candidate genes by their differentially expressed neighborhoods. We have developed an efficient algorithm to compute a genomewide network by applying the Cholesky decomposition. To illustrate the power of our method, we have applied it to four constitutional genetic disorders and successfully prioritized the known disease-causing genes. In an application on a genetic disorder that is not yet well studied, we could retrieve two important known candidate genes and suggest a new candidate gene.

## Materials and Methods

### Overview

A network model based on the STRING database [8] was built for human genes. This sparse network model was used to compute distances between single genes via the Laplacian Exponential Diffusion Kernel [36]. The goal of such an approach is to build a network model based on global similarity measure (i.e., to include both direct and indirect connections between genes).

For each disease, a set of candidate genes (e.g., from a linkage study) has to be defined and a relevant microarray experiment is required (e.g., diseased sample vs. reference sample). A main issue that arises when applying kernel graph methods to genomewide networks is the computing time. To reduce it, the kernel matrices were approximated by the full or incomplete Cholesky decomposition and the reduced eigenvalue decomposition. Knowing the distances between single genes in the network, the differential expression level of adjacent genes can be considered. Each candidate gene receives a score based on the analysis of the differentially expressed neighborhoods, by which it is ranked. Candidate genes with genes in their neighborhood having highly differentially expressed indices are strong candidates.

### Functional protein association network

A protein association network is an undirected graph with proteins as nodes and weights as edges. If there exists an association between two proteins, an edge will be set between the corresponding nodes in the graph. The weights of the edges are taken represent the probability that such an association exists in reality. To model our functional protein association network, we used data from STRING [8] (version 7.1). STRING is a database of known and predicted protein-protein interactions. The interactions are derived from different information sources and different organisms, whereas the interactions include physical and functional associations. To build our networks, we have used the fused network provided by STRING that already integrates all available protein-protein interactions.

### Kernel matrix and distance network

We hypothesize that global network-similarity measures capture relationships between disease proteins better than algorithms based on direct interactions. To capture global relationships within a graph, a graph kernel was used. A graph kernel computes the global similarity of two nodes as the probability of reaching one node at some time point after a random walk starting from another node. This global similarity detects, besides direct, also indirect connections in the graph. The resulting graph leads to a global distance network where the edge between two nodes does not represent a direct interaction, but rather the global distance in this network.

The **Laplacian Exponential Diffusion Kernel** was introduced by *Kondor and Lafferty (2002)* [36] as
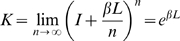
(1)whereby *L* is the Laplacian matrix of a weighted and undirected graph *G* with symmetric weights [36], [37], and the parameter *β* is the diffusion parameter that determines the degree of diffusion (for more details see Supplementary Materials). For a Laplacian matrix, 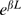 is always positive definite and thus can be used as a kernel matrix. It can be seen as a random walk, starting from a node and transitioning to a neighboring node with the probability *β*.

The complexity of Equation (1 is 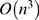. To improve the performance of the computation of such a diffusion, we can apply the Cholesky decomposition, that is decomposing the Laplacian matrix into the product of a lower triangular matrix by its transpose, before computing the kernel from this matrix. In this way, the computing time is reduced significantly as presented in Table S7.

The resulting kernel matrix is a densely connected network. This new network detects not only the direct interaction from the original protein association network, but also all indirect interactions via other genes. Thanks to this property the distances between all genes in the network can be determined. They are necessary for identifying highly expressed neighborhoods with a certain distance from a candidate gene, even if the genes are not directly interacting.

### Cholesky decomposition

There exists for every symmetric positive definite matrix *A* of rank *n* exactly one lower triangular matrix 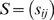 with 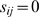 if 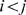 and 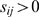 if *i* = 1, 2,…, *n*.

The Cholesky decomposition (CD) solves the linear equation 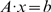 by transforming *A* into a product of a lower triangular matrix *S* of rank *n* and its transposed 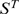:
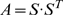
(2)To reduce the dimensionalities of kernel matrices we applied the Incomplete Cholesky decomposition (ICD) with pivoting [40]–[42].

ICD is an iterative algorithm that approximates a lower rank matrix (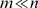) in order to reduce the dimensionalities so that
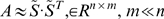
(3)The resulting matrix 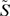 is a lower triangular matrix of rank *m*. The overall complexity is 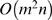 and the storage requirement is 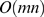.

### Distance network

Having a symmetric and positive semidefinite Laplacian matrix L, we can compute the Laplacian Exponential Diffusion Kernel by applying CD or ICD instead of Equation (1), the computing time can be reduced significantly from 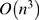 to 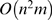, in which *m* is the rank of *S* (Equations 2–3)).

In Table S7, we show that the computing time in calculating the Laplacian Exponential Diffusion Kernel can be significantly reduced by applying the CD. If we can accept an approximation to the exact kernel, we can apply the ICD by defining a threshold to reduce the rank of the matrices. In our example, we chose a threshold that led to an error of 7%–10%, depending on the size of the matrix (Table S8). The error is the difference of the norm of the reduced rank matrix and of the full rank matrix. For a network of the whole human genome containing 16,566 genes and an error of 7%, the resulting matrix got a low rank matrix with a rank of 1,829 that could be computed in 35 minutes, whereas by running the computation by applying the initial equation introduced by *Kondor and Lafferty (2002)* [36] (Equation (1)), the computation on the same machine (dual Opteron 250 with 16 GB RAM) ran out of memory and could not finish the computation. Even if no error can be handled and the CD is applied, and thus the matrices remain full rank, the computation could be successfully finished after 32 hours.

### Gene expression analysis

After obtaining a global distance network by computing the Laplacian Exponential Diffusion Kernel from the protein association network, the gene expression profiles for a specific disease can be mapped onto this network. For this purpose, relevant genome-wide microarray experiment data (e.g., disease sample vs. reference sample) is required from which the differential expression level for each gene in the genome is computed. Basing on the microarray experiment (the datasets that we chose from GEO were from good quality and already normalized) the fold change between the two conditions can be computed for each gene. We applied the fold-change between control and experiment [39]. For our method only the absolute value of the fold-change is relevant (i.e., if a gene is highly differentially expressed or not). We do not use a threshold to distinguish between highly and lowly differentially expressed genes, because our method considers all differential expression levels for computing the scores.

### Scoring and ranking

**Figure 3:**
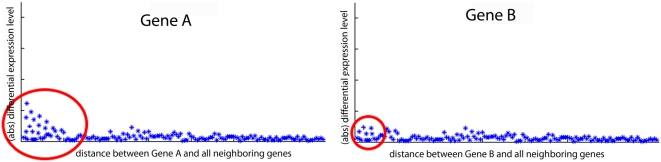
Level of differential expression as function of distance from the candidate for two different candidate genes. *Gene A* has a more differentially expressed genes among its close neighboring genes in the distance network than *gene B*, and is therefore a better candidate gene than *gene B*.

For each candidate gene a score is computed based on the differential expression levels of its neighborhood. For this purpose the differential expression levels of all neighbors in the distance network are ordered by their distance to the candidate gene. The rank of the diffusion distance is then taken as the new distance measure. The differential expression levels are then multiplied by a weighting function (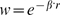, controlled by parameter *β* and rank *r*) to stress expression of close neighbors and to suppress expression of neighbors being far. Then the expression data is distributed randomly over the network. The score for a candidate gene is defined by the maximum deviation between its weighted neighboring expression and the randomized expression. Thus, the higher the level of differential expression level of close neighboring genes, the higher the score for the candidate gene (see Figure 3).

To obtain an empirical p-value for each candidate gene, the distribution of the scores was determined by repeatedly (3,000 times) randomly distributing the expression data over the network to estimate the significance of the signals of the actual candidates. By comparing the score of each candidate gene with the random distribution of the scores, a *p*-value was assigned. If a candidate's score was larger than 95% (α = 0.05) of all randomized scores, this candidate gene's score was considered significant.

### Neighborhood size

For our soft neighborhoods, we also define a notion of *neighborhood size*. Instead of having a hard threshold on the distance that determines whether a gene belongs to the neighborhood or not, our weighting function has a scale parameter that determines how quickly the weight decreases as a function of the distance. This parameter is what defines our neighborhood “size”. To communicate about the neighborhood size easily, we use the following procedure. First, a threshold on the weight is selected, meaning that we neglect the contribution of all genes that have a weight lower than the threshold and are thus sufficiently “far away” from the candidate (the number of considered genes varies between 20 and 4000, depending on *β* that varies from 0.001 and 0.5 (see Table S9)). As a result, we view the genes for which the weight is considered nonnegligible as “inside” the neighborhood. In our case, the weighting function is a negative exponential function (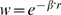) regulated by a parameter *β*. As *β* decreases, the neighborhood size increases, and more neighboring genes are considered for computing the scores of the candidates (see Table S9).

For each data set, an appropriate neighborhood size must be computed independently. Therefore, we first run the analysis for various value of *β* (from 0.001 to 0.5) and, for each, measure the signal captured by using the Fisher omnibus statistic (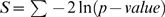) on the rankings produced. We then generate a new *p*-value from the statistic *S* for each *β* using the χ^2^ distribution. The value of the parameter *β* with the smallest corresponding *p*-value is considered the appropriate neighborhood size for this data set. The idea is that, for an appropriate neighborhood size, some of the candidates will capture meaningful signal from their neighbors. By contrast, for inappropriate neighborhood sizes, all candidates will have uniformly distributed *p*-values, leading to a low statistic *S*.

Table S9 illustrates the signals derived by the Fisher omnibus meta-analysis using the example of FXS [11], leading to an appropriate neighborhood size of approximately 150 genes for this data set.

### Data sets

- FXS
  - Mendelian disorder: fragile X syndrome (FXS, OMIM #300624)
  - Disease gene: fragile X mental retardation 1 (FMR1, OMIM *309550)
  - Phenotype: mental retardation, macroorchidism, and distinct facial features
  - Expression data: *Nishimura et al. (2007)* [11] (GEO accession number: GSE7316). Lymphoblastoid cell cultures from patients with confirmed FMR1 full mutation (CGG repeat expansion). Platform: Agilent-012391 Whole Human Genome Oligo Microarray G4112A
- MFS
  - Mendelian disorder: Marfan syndrome (MFS, OMIM #154700)
  - Disease gene: fibrillin-1 precursor (FBN1, OMIM *134797)
  - Phenotype: variable skeletal abnormalities, tall stature, disproportionately long limbs and digits, joint laxity, eye anomalies and progressive cardiovascular problems.
  - Expression data: *Yao et al. (2007)* [12] (GEO accession number GDS2960). Fibroblast cultures from patients with confirmed FBN1 missense (9) and nonsense (7) mutations as well as one multi-exon deletion. Platform: Research Genetics (Invitrogen) - GF211 Microarray Filter
- CF
  - Mendelian disorder: Cystic fibrosis (CF, OMIM #219700)
  - Disease gene: Cystic fibrosis transmembrane conductance regulator (CFTR, OMIM *602421, [43])
  - Phenotype: chronic obstructive lung disease, bronchiectasia, and exocrine pancreatic insufficiency
  - Expression data: *Wright et al. (2006)* [13] (GEO accession number GDS2143). Analysis of the nasal respiratory epithelium of cystic fibrosis (CF) patients with mild (4) or severe (5) lung disease. Platform: Affymetrix GeneChip Human Genome U133 Array Set HG-U133B
- BMD
  - Mendelian disorder: Becker muscular dystrophy (BMD, OMIM #300376, [44])
  - Disease gene: dystrophin (DMD, OMIM *300377)
  - Phenotype: muscle wasting and weakness, and in some cases with mental impairment.
  - Expression data: *Bakay et al. (2006)* [14] (GEO accession number GDS2855). Analysis of muscle biopsy specimens from patients with various muscle diseases. Platform: Affymetrix GeneChip Human Genome U133 Array Set HG-U133B
- Stein-Levental syndrome
  - Mendelian disorder: Stein-Levental syndrome ( OMIM %184700)
  - Putative disease genes: follistatin (FST (HGNC symbol), OMIM *136470) putatively related with Stein-Levental syndrome [24], fibrillin 3 (FBN3), OMIM *608529) putatively associated with Stein-Levental syndrome [26].
  - Phenotype: obesity, hyperandrogenism and chronic anovulation
  - Expression data: *Cortón et al. (2007)* [22] (GEO accession number: GDS2084). Omental fat biopsy from patients. Unconfirmed disorder etiology. Platform: Affymetrix GeneChip Human Genome U133 Array Set HG-U133A

## Supporting Information

Supplementary Materials S1 (0.07 MB DOC)

Figure S1 Neighborhood of FMR1 and DUSP9. These graphs show the closest neighbors of FMR1 (A) and DUSP9 (B) including their differential expression levels and distances to FMR1 and DUSP9. For genes with absolute differential expression levels (2-fold-changes) larger than 1.5, the nodes are labeled with the gene names, the node size increases with the value and the color gets darker. With decreasing distances (i.e., increasing similarities) the edges between FMR1 or DUSP9 and their neighbors become thicker. This picture shows that the neighborhood of FMR1 belongs to a more disrupted expression module than the neighborhood of DUSP9.
(2.11 MB TIF)

Figure S2 Neighborhood of FBN1 and LEO1. These graphs show the closest neighbors of FBN1 (A) and LEO1 (B) including their differential expression levels and distances to FBN1 and LEO1. For genes with large absolute differential expression levels (2-fold-changes), the nodes are labeled with the gene names, the node size increases with the value and the color gets darker.With decreasing distances (i.e., increasing similarities) the edges between FBN1 or LEO1 and their neighbors become thicker. This picture shows that the neighborhood of FBN1 belongs to a more disrupted expression module than the neighborhood of LEO1.
(2.43 MB TIF)

Figure S3 Neighborhood of CFTR and PIK3CG. These graphs show the closest neighbors of CFTR (A) and PIK3CG (B) including their differential expression levels and distances to CFTR and PIK3CG. For genes with large absolute differential expression levels (2-fold-changes), the nodes are labeled with the gene names, the node size increases with the value and the color gets darker. With decreasing distances (i.e., increasing similarities) the edges between CFTR or PIK3CG and their neighbors become thicker. This picture shows that the neighborhood of CFTR belongs to a more disrupted expression module than the neighborhood of PIK3CG.
(2.22 MB TIF)

Table S1 Top 25 ranked candidate genes in Fragile X syndrome (FXS). Fragile X syndrome [11] is a disorder caused by mutation in the FMR1 gene, and is characterized by mental retardation, macroorchidism, and distinct facial features. Candidate genes were chosen from chrXq26-q28 that contains 119 genes including FMR1. These candidate genes were ranked by our new approach, and the top 25 ranked candidate genes are presented here, whereas the top eleven genes have significant p-values (α = 0.05). FMR1 ranked first with a significant p-value (0.00131), and FMR2, also involved in FXS, got a significant p-value of 0.01991 on position 6. Out of the eleven significant candidate genes in the ranking we identified four genes, including FMR1 and FMR2, that are known to be linked to mental retardation [11], [15]–[17].
(0.06 MB DOC)

Table S2 Top 25 ranked candidate genes in Marfan syndrome (MFS). Marfan syndrome [12] is a heritable connective tissue disorder caused by mutations in the FBN1 gene, and is characterized by increased height, disproportionately long limbs and digits, anterior chest deformity, joint laxity, vertebral column deformity, and other variable skeletal abnormalities, as well as several ocular and cardiovascular features. Candidate genes were chosen from 15q15.3-q22.33 that contains 129 genes including FBN1. These candidate genes were ranked by our new approach, and the top 25 ranked candidate genes are presented here, whereas the top six genes have significant p-values (α = 0.05). FBN1 was ranked on the fifth position with a significant p-value (0.0226). In the ranking we obtained six genes that were significant but not involved in MFS or phenotype related diseases.
(0.06 MB DOC)

Table S3 Top 25 ranked candidate genes in Cystic fibrosis (CF). Cystic fibrosis [13] is an autosomal recessive disorder of epithelial ion transport caused by mutations in the CF transmembrane conductance regulator gene (CFTR), and is characterized by chronic obstructive lung disease, bronchiectasia, and exocrine pancreatic insufficiency. Candidate genes were chosen from chr7q22.1-31.33 that contains 110 genes including CFTR. These candidate genes were ranked by our new approach, and the top 25 ranked candidate genes are presented here, whereas the top nine genes have a significant p-value (α = 0.05). CFTR was ranked in the seventh position with a significant p-value (0.046). In the ranking we obtained seven genes that were significant but not involved in CF or phenotype related diseases. However, out of the top 25 ranked genes we detected four genes that are known to be linked to CF [18]–[21].
(0.06 MB DOC)

Table S4 Top 25 ranked candidate genes in Becker muscular dystrophy (BDM). Becker muscular dystrophy [14] is a X-linked progressive myopathy caused by mutations within the DMD gene, and is characterized by muscle wasting and weakness, and in some cases with mental impairment. Candidate genes were chosen from chrXp22.33-21.1 that contains 116 genes including DMD. These candidate genes were ranked by our new approach, and the top 25 ranked candidate genes are presented here, whereas the top two genes have significant p-values (α = 0.05). DMD ranked on the second position with a significant p-value (0.0272). The other significant gene is not involved in BMD or in a phenotype related disease.
(0.06 MB DOC)

Table S5 Top 25 ranked candidate genes from chr5q11.2 in Stein-Levental syndrome. Stein-Levental syndrome [22] is a oligogenic hormonal disorder among women putatively related with the FST gene, and is characterized by hyperandrogenism, chronic anovulation and associated with obesity. Candidate genes were chosen from chr5q11.2 that contains 25 genes including the candidate gene FST [24]. These candidate genes were ranked by our new approach, whereas only the top gene has a significant p-value (α = 0.05). FST was ranked in the second position with a p-value of 0.056, and we received only one significant gene (DEAD box 4) that is a plausible candidate gene for Stein-Levental syndrome.
(0.06 MB DOC)

Table S6 Top 25 ranked candidate genes from chr19p13.2 in Stein-Levental syndrome. Stein-Levental syndrome [22] is a oligogenic hormonal disorder among women putatively associated with the FBN3 gene, and is characterized by hyperandrogenism, chronic anovulation and associated with obesity. Candidate genes were chosen from chr19p13.2 that contains 100 genes including the candidate gene FBN3 [26]. These candidate genes were ranked by our new approach, and the top 25 ranked candidate genes are presented here, whereas only the top gene has a significant p-value (α = 0.05). FBN3 was ranked in the fifth position with a p-value of 0.0595.
(0.06 MB DOC)

Table S7 CPU times for computing the Laplacian Exponential Diffusion Kernel by its definition, the CD and the ICD. CPU times (sec) for computing the Laplacian Exponential Diffusion Kernel by its definition (Equation 1), by CD (Equation 2), and by ICD (Equation 3) with a threshold leading to an error of 7%–10%. The computation were run on a dual Opteron 250 with 16 GB RAM.
(0.03 MB DOC)

Table S8 Rank and Error of resulting Laplacian Exponential Diffusion Kernel computed by its definition, the CD and the ICD. Rank and Error of resulting Laplacian Exponential Diffusion Kernel computed by its definition, (Equation 1), by CD (Equation 2), and by ICD (Equation 3) with a threshold leading to an error of 7%–10%. The computation were run on a dual Opteron 250 with 16 GB RAM.
(0.03 MB DOC)

Table S9 Example for determining an appropriate neighborhood size using the example of data set 1 (FXS [11]). The neighborhood size is controlled by a weighting function (w = exp(−β⋅r). Applying the Fisher omnibus meta-analysis (S = ∑−2 ln (p-value)) for each parameter β, new p-values are generated from a Χ∧2 distribution. The parameter β , for which the smallest p-value is observed (here: β = 0.05), leads to the appropriate neighborhood size for FXS (approx. 150 genes).
(0.02 MB DOC)
