# Supplementary material for: Network Analysis of Differential Expression for the Identification of Disease-Causing Genes
Source: PLoS One. 2009 May 13;4(5):e5526. doi: 10.1371/journal.pone.0005526 (PMC2677677; doi:10.1371/journal.pone.0005526)
Supplement: Supplementary Materials S1 — (0.07 MB DOC) [file pone.0005526.s001.doc]

## Case Studies

### Case Study 1: Fragile X syndrome (FXS)

The first data set we ran with our method was a microarray analysis to compare the mRNA expression profile in lymphoblastoid cells from patients with autism due to a fragile X mutation (FMR1-FM) and non-autistic controls [11]. Fragile X syndrome (FXS) is a disorder caused by expansion of the CGG trinucleotide repeat in the 5’ untranslated region of the fragile X mental retardation 1 (FMR1) gene gene located on chrXq27.3, and is characterized by mental retardation, macroorchidism, and distinct facial features.

Candidate genes were chosen from chrXq26-q28 that contains 119 genes including FMR1. The top 25 ranked genes are presented in Table S1.

**Neighborhood**

For the ranking of FXS a neighborhood of 150 genes was determined, i.e. we have considered the 150 genes with the smallest distances to the candidate genes, because larger neighborhoods were found to be meaningless for this ranking due to large changes in the expression for very close neighboring genes (data not shown).

Comparing the disrupted expression neighborhoods of FMR1 and the low ranked gene DUSP9 (see Table S1), a graph containing up to the nearest 100 genes could be drawn (see Figure S1). This visualization shows that the closest neighborhood of FMR1 is higher differentially expressed than of DUSP9, and that the neighborhood of FMR1 belongs to a more disrupted expression module than the neighborhood of DUSP9.

**Significance**

Figure 2-A shows the distribution of p-values after 10,000 randomizations, and the p-value of FMR1 which is significant. This figure illustrates that there are only few genes having significant p-values after a randomization procedure, and that the high rank of FMR1 is not random.

**Ranking**

FMR1 ranked first with a significant p-value (0.001), and FMR2, also involved in mental retardation, got a significant p-value of 0.02 on rank 6 out of 119 ranked genes.

Out of the eleven significant candidate genes in the ranking we identified four genes, including FMR1 and FMR2 [15] that are known to be linked to mental retardation.

Mutation in the creatine transporter gene SLC6A8 causes the X-linked Creatine Deficiency syndrome, a mental retardation disease [16].

Mutations in the MECP2 gene cause Rett syndrome, a severe neurodevelopmental disorder including mental retardation (e.g. [17]).

Summarizing, four of the eleven significant genes in our ranking are directly associated with mental retardation.

### Case Study 2: Marfan syndrome (MFS)

The second data set we ran with our method was a microarray analysis to compare the mRNA expression profile in cultured skin fibroblasts from patients with Marfan syndrome (MFS) and controls [12]. MFS is a heritable autosomal dominant connective tissue disorder caused by mutations in the FBN1 gene, and is characterized by variable skeletal abnormalities, tall stature, disproportionately long limbs and digits, joint laxity, eye anomalies and progressive cardiovascular problems.

Candidate genes were chosen from 15q15.3-q22.33 that contains 129 genes including FBN1. Their top 25 ranked genes are presented in Table S2.

**Neighborhood**

For the ranking of MFS a neighborhood of again 150 genes was determined, because the largest disruptions in expressed neighborhoods could be again observed in small neighborhoods (data not shown).

Comparing the disrupted expression neighborhoods of FBN1 and the low ranked gene LEO1 (see Table S2), a graph containing up to the nearest 100 genes could be drawn (see Figure S2). This visualization shows that the neighborhood of FBN1 belongs to a more disrupted expression module than the neighborhood of LEO1 because of further highly differentially expressed genes in its neighborhood.

**Significance**

Figure 2-B shows the distribution of p-values after 10,000 Randomizations, and the p-value of FBN1 which is significant. This figure illustrates that there are only few genes having significant p-values after a randomization procedure, and that the high rank of FBN1 is not random.

**Ranking**

FBN1 was ranked in the fifth position out of 129 ranked candidate genes with a significant p-value (0.02). In the ranking we obtained six genes that were significant but not involved in MFS or phenotype related diseases.

### Case Study 3: Cystic fibrosis (CF)

The third data set we ran with our new method was a high-density oligonucleotide microarray analysis of nasal respiratory epithelium tissues of patients with cystic fibrosis (CF) [13**]**. Cystic fibrosis is an autosomal recessive disorder of epithelial ion transport caused by mutations in the CF transmembrane conductance regulator gene (CFTR), and is characterized by chronic obstructive lung disease, bronchiectasia, and exocrine pancreatic insufficiency.

Candidate genes were chosen from chr7q22.1-31.33 that contains 110 genes including CFTR. Their ranking is presented in Table S3.

**Neighborhood**

For the ranking of CF a neighborhood of 20 genes was determined, because differences in the expression could be already detected for small neighborhoods for the top ranked genes (data not shown), and an expansion of the neighborhood was not necessary.

Comparing the disrupted expression neighborhoods of CFTR and the low ranked gene PIK3CG (see Table S3), a graph containing 100 neighboring genes could be drawn (see Figure S3). This visualization shows that the higher ranked gene CFTR belongs to a more disrupted expression module than PIK3CG.

**Significance**

Figure 2-C shows the distribution of p-values after 10,000 Randomizations, and the p-value of CFTR which is significant and ranked on the seventh position. This figure illustrates that there are only few genes having significant p-values after a randomization procedure, and that the high rank of CFTR is not random.

**Ranking**

CFTR was ranked in the seventh position out of 110 ranked candidate genes with a significant p-value of 0.04. In the ranking we obtained seven genes that were significant but not involved in CF or phenotype related diseases. However, out of the top 25 ranked genes we detected four genes that are known to be linked to CF.

The SLC26 gene family, including the top ranked genes SLC26A4 and SLC26A5, encodes anion exchangers. SLC26 Cl(-)-HCO(3)(-) and Cl(-)-OH(-) exchange is activated by the CFTR gene implicating defective regulation of these exchangers in the reduced HCO(3)(-) transport seen in CF [18].

By microarray analysis, [19] found coordinated downregulation of mucins, including MUC17, in colon of patients with inflammatory bowel disease, including Crohn disease and ulcerative colitis, compared with controls. [20] found an increased expression of Muc3 (orthologue of human MUC17) in a in the small intestine of CF mice.

[21] reported an association of distinct variants of the intestinal mucin gene MUC3A with ulcerative colitis and Crohn's disease.

Summarizing, four of the top 25 ranked genes are directly associated with CF or a disease related phenotype.

### Case Study 4: Becker muscular dystrophy (BMD)

The fourth data set we ran with our new method was a microarray analysis of muscle biopsy specimens from patients with various muscle diseases (including BMD) [14]. Becker muscular dystrophy is a X-linked progressive myopathy caused by mutations within the DMD gene, and is characterized by muscle wasting and weakness, and in some cases with mental impairment.

Candidate genes were chosen from chrXp22.33-21.1 that contains 116 genes including DMD. The top 25 ranked genes are presented in Table S4.

**Neighborhood**

For the ranking of BMD a neighborhood of 2000 genes was determined, because large differences in the expression could be detected only for large neighborhoods for the top ranked genes, whereas for smaller neighborhoods the top ranked genes had no significant difference, thus, the neighborhood had to be expanded (data not shown).

**Significance**

Figure 2-D shows the distribution of p-values after 10,000 Randomizations, and the p-value of DMD which is significant and ranked on the second position. This figure illustrates that there are only few genes having significant p-values after a randomization procedure, and that the high rank of CFTR is not random.

**Ranking**

DMD ranked on the second position out of 116 ranked candidate genes with a significant p-value (0.02). In the ranking we obtained two genes that were significant, including DMD. The other significant gene is not involved in BMD or in a phenotype related disease.

### Case Study 5: Stein-Levental syndrome

The fifth data set we ran with our new method was a microarray analysis of omental adipose tissues of morbidly obese patients with Stein-Levental syndrome [22]. Stein-Levental syndrome is a hormonal disorder among women characterized by obesity, hyperandrogenism and chronic anovulation, and is putatively associated with mutation of FST (follistatin)) [24] or FBN3 (fibrillin 3) [26].

Candidate genes were chosen from chr5q11.2 that contains 25 genes including FST, and from chr19p13.2 that contains 100 genes including FBN3. Their rankings are presented in Tables S5-S6.

**Neighborhood**

For the ranking of Stein-Levental syndrome a neighborhood of 2000 genes was determined, because large differences in the expression could be detected only for large neighborhoods for the top ranked genes, whereas for smaller neighborhoods the top ranked genes had no significant difference, thus, the neighborhood had to be expanded (data not shown).

**Ranking**

FST was ranked in the second position out of 25 candidate genes with a p-value of 0.056 (Table S5), and we received only one significant gene (DEAD box 4) that is a plausible candidate gene for Stein-Levental syndrome.

FBN3 was ranked on the fifth position out of 100 candidate genes with a p-value of 0.0595 (Table S6).

**Kernel matrix – Distance Network**

Consider a given weighted and undirected graph *G* with symmetric weights $w_{ij}>0$ between all linked couple of nodes *i* and *j*. The weight $w_{ij}>0$ increases with the importance of the relation between nodes *i* and *j*: the larger its value, the easier the communication through the edge. The Adjacency matrix *A* is defined as $a_{ij}=w_{ij}$if the nodes *i* and *j* are directly connected and $a_{ij}=0$otherwise. The Laplacian matrix *L* of *G* is defined as $L=Diag(a_{i.})-A$, with the diagonal degree matrix $Diag(a_{i.})$ whereby its entries are defined as $d_{ii}=a_{i.}=\sum_{j=1}^{n} a_{ij}$ [36,37].

The **Laplacian Exponential Diffusion Kernel** was introduced by *Kondor and Lafferty (2002)* [36] as

$K=\lim_{n\to\infty} \left(I+\frac{\beta L}{n}\right)^{n}=e^{\beta L}$ (1)

whereby the parameter *β* is the diffusion parameter that determines the degree of diffusion. For a Laplacian matrix, $e^{\beta L}$is always positive definite and thus can be used as a kernel matrix. The diffusion effect of the kernel can be illustrated by

$e^{\beta L}=I+\sum_{n=1}^{\infty} \left(\frac{\beta^{n}}{n!}L^{n}\right)$ (2)

The kernel matrix *K* can be seen as a random walk, starting from a node and transitioning to a neighboring node with the probability *β*. Fixing the transition probability to every neighboring node as *β,* the probability of staying at the current node *i* is $1-d_{i}\beta$, where $d_{i}$is the degree of node *i*. The column vector *j* of *K* then represents the steady-state probability vector of the random walk when starting at node *j*, and the value$K_{ij}$represents the probability that a random walk starting from *i* will be at *j* after infinite time steps. If the diffusion parameter is small, *K* can be seen as a lazy random walk because the probability that the random walker stays at the current node is high. As *β* increases, the kernel values diffuse more completely through the graph, and when *β* is sufficiently large, the values among distant nodes capture the long-range relationships between nodes [36].

Beside the direct protein-protein interaction, the connectivity of the starting and ending node in a random walk play a central role in determining the global similarity in an interaction network.

The computation of a kernel matrix after Equation (1) is very time intensive for large networks, and its complexity is$O(n^{3})$. If this kernel is computed for a whole genome, the computing time and the storage requirement must be improved. This can be reached by applying the Cholesky decomposition by decomposing the Laplacian matrix into a lower triangular matrix before computing the kernel from this matrix. By doing so, the computing time could be reduced significantly as presented in Table S7.
