# Supplementary figures and images for: Network Analysis of Differential Expression for the Identification of Disease-Causing Genes
Source: PLoS One. 2009 May 13;4(5):e5526. doi: 10.1371/journal.pone.0005526 (PMC2677677; doi:10.1371/journal.pone.0005526)

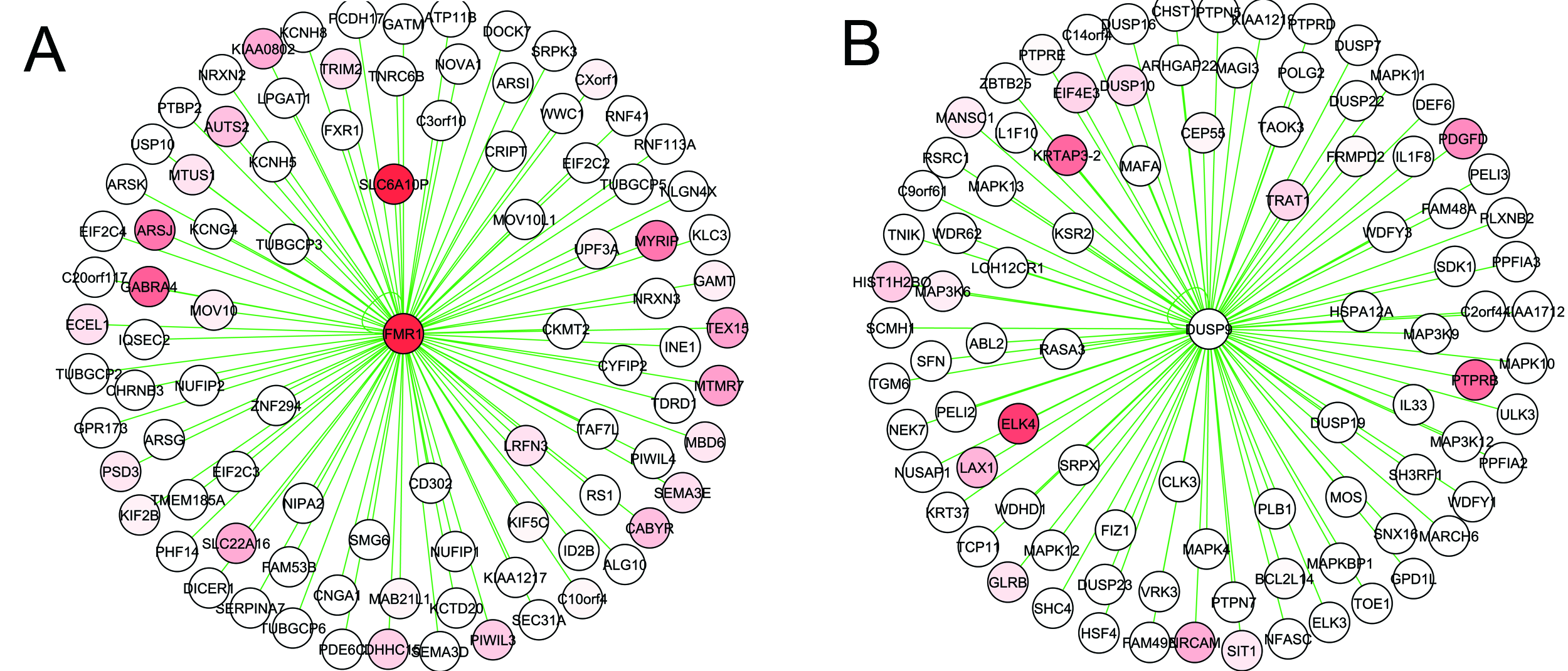

Supplement: Figure S1 — Neighborhood of FMR1 and DUSP9. These graphs show the closest neighbors of FMR1 (A) and DUSP9 (B) including their differential expression levels and distances to FMR1 and DUSP9. For genes with absolute differential expression levels (2-fold-changes) larger than 1.5, the nodes are labeled with the gene names, the node size increases with the value and the color gets darker. With decreasing distances (i.e., increasing similarities) the edges between FMR1 or DUSP9 and their neighbors become thicker. This picture shows that the neighborhood of FMR1 belongs to a more disrupted expression module than the neighborhood of DUSP9. (2.11 MB TIF) [file pone.0005526.s002.tif]

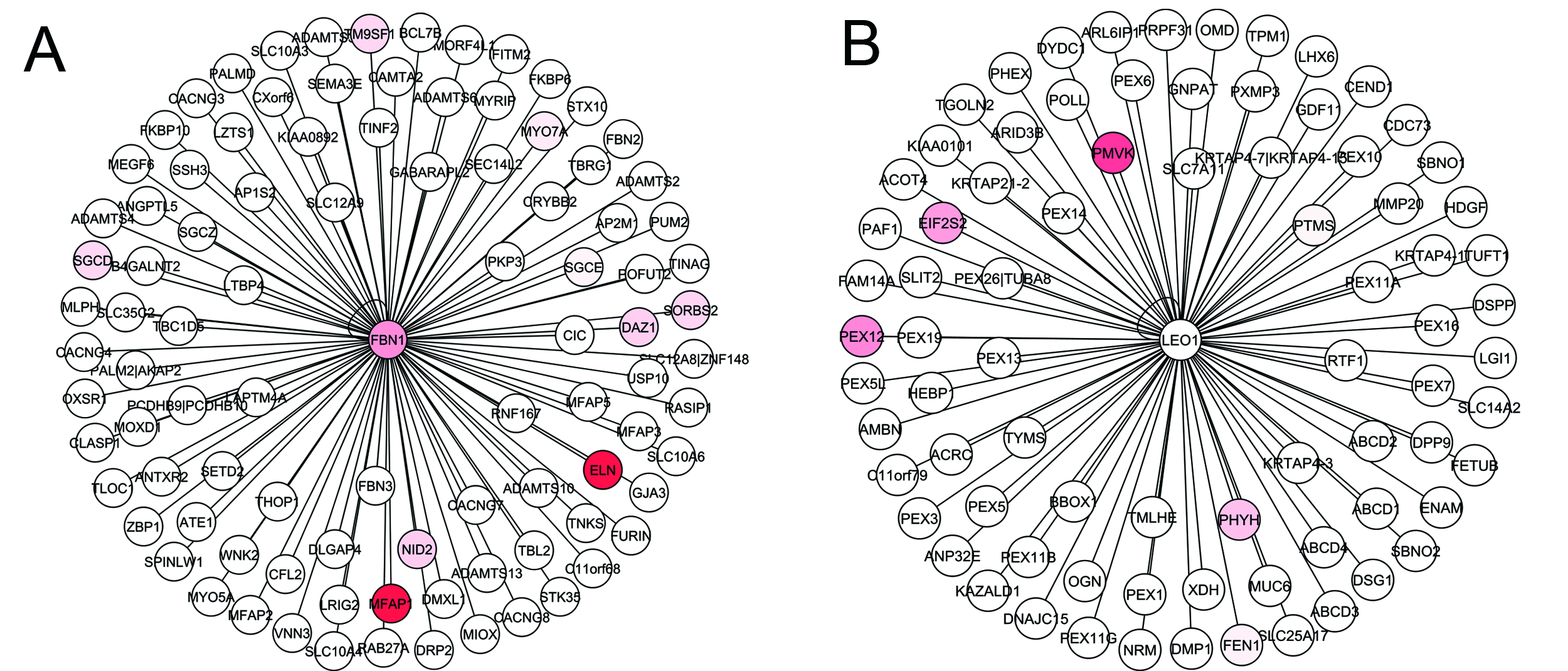

Supplement: Figure S2 — Neighborhood of FBN1 and LEO1. These graphs show the closest neighbors of FBN1 (A) and LEO1 (B) including their differential expression levels and distances to FBN1 and LEO1. For genes with large absolute differential expression levels (2-fold-changes), the nodes are labeled with the gene names, the node size increases with the value and the color gets darker.With decreasing distances (i.e., increasing similarities) the edges between FBN1 or LEO1 and their neighbors become thicker. This picture shows that the neighborhood of FBN1 belongs to a more disrupted expression module than the neighborhood of LEO1. (2.43 MB TIF) [file pone.0005526.s003.tif]

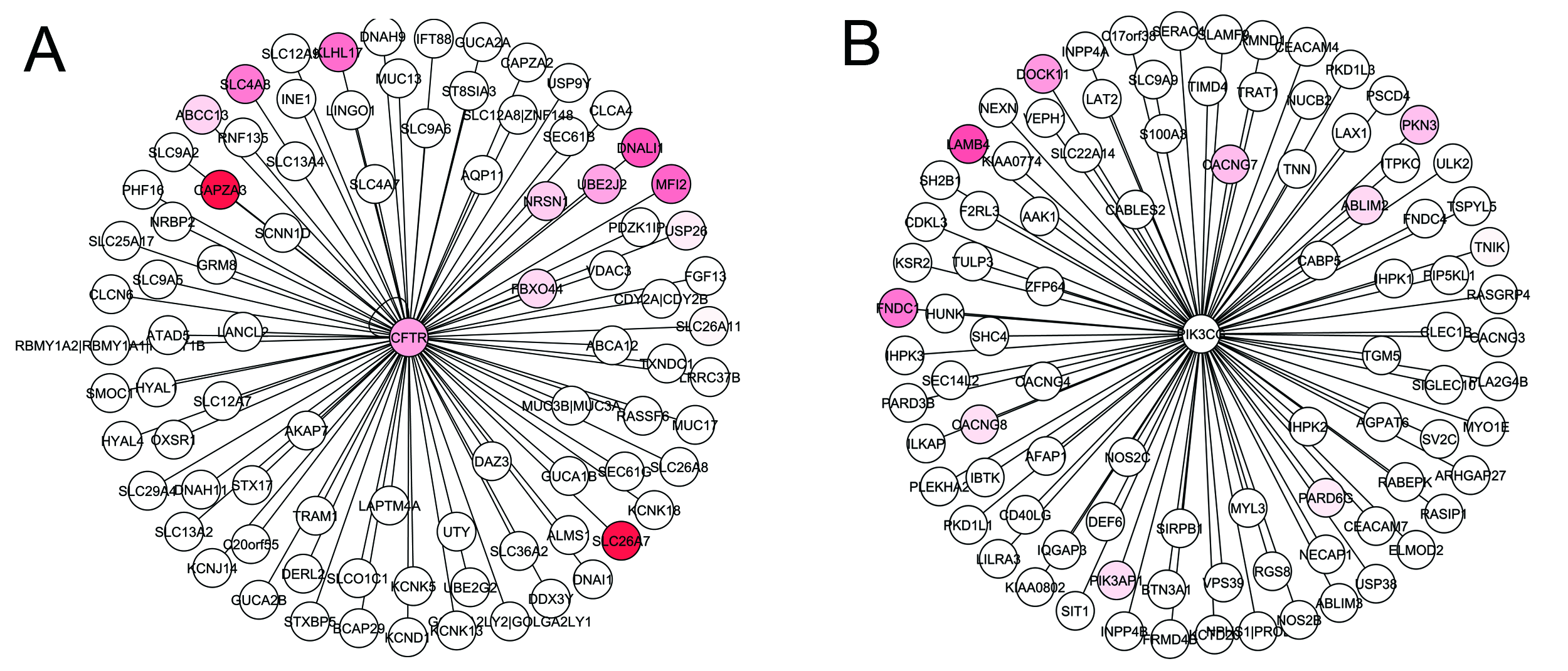

Supplement: Figure S3 — Neighborhood of CFTR and PIK3CG. These graphs show the closest neighbors of CFTR (A) and PIK3CG (B) including their differential expression levels and distances to CFTR and PIK3CG. For genes with large absolute differential expression levels (2-fold-changes), the nodes are labeled with the gene names, the node size increases with the value and the color gets darker. With decreasing distances (i.e., increasing similarities) the edges between CFTR or PIK3CG and their neighbors become thicker. This picture shows that the neighborhood of CFTR belongs to a more disrupted expression module than the neighborhood of PIK3CG. (2.22 MB TIF) [file pone.0005526.s004.tif]
