# Supplementary material for: Network Analysis of Differential Expression for the Identification of Disease-Causing Genes
Source: PLoS One. 2009 May 13;4(5):e5526. doi: 10.1371/journal.pone.0005526 (PMC2677677; doi:10.1371/journal.pone.0005526)
Supplement: Table S1 — Top 25 ranked candidate genes in Fragile X syndrome (FXS). Fragile X syndrome [11] is a disorder caused by mutation in the FMR1 gene, and is characterized by mental retardation, macroorchidism, and distinct facial features. Candidate genes were chosen from chrXq26-q28 that contains 119 genes including FMR1. These candidate genes were ranked by our new approach, and the top 25 ranked candidate genes are presented here, whereas the top eleven genes have significant p-values (α = 0.05). FMR1 ranked first with a significant p-value (0.00131), and FMR2, also involved in FXS, got a significant p-value of 0.01991 on position 6. Out of the eleven significant candidate genes in the ranking we identified four genes, including FMR1 and FMR2, that are known to be linked to mental retardation [11], [15]–[17]. (0.06 MB DOC) [file pone.0005526.s005.doc]

| **Rank** | **Symbol** | **Score** | **2fold-change** | **p-value** | **Linkage to phenotype** |
| --- | --- | --- | --- | --- | --- |
| **1** | **FMR1** | **0.0028** | **4.81** | **0.0013** | **FXS [11,15]** |
| 2 | FAM11A | 0.0023 | 0 | 0.0036 |  |
| 3 | SLC6A8 | 0.0016 | 0.13 | 0.0105 | X-linked mental retardation [16] |
| 4 | MAGEA9 | 0.0015 | 2.44 | 0.0164 |  |
| 5 | MPP1 | 0.0015 | 2.75 | 0.0169 |  |
| **6** | **FMR2** | **0.0014** | **1.08** | **0.0199** | **FXS** [15] |
| 7 | G6PD | 0.0013 | 0.60 | 0.0289 |  |
| 8 | BRS3 | 0.0012 | 0.35 | 0.0365 |  |
| 9 | SPANXA2 | 0.0012 | 0 | 0.0419 |  |
| 10 | H2AFB2 | 0.0012 | 0.35 | 0.0421 |  |
| 11 | MECP2 | 0.0011 | 1.10 | 0.0479 | Rett Syndrome [17] |
| 12 | ZNF185 | 0.0010 | 2.71 | 0.0699 |  |
| 13 | MAGEC2 | 0.0010 | 1.09 | 0.0699 |  |
| 14 | GAB3 | 0.0010 | 1.12 | 0.0721 |  |
| 15 | TEX28 | 0.0010 | 0 | 0.0723 |  |
| 16 | PLAC1 | 0.0010 | 0.40 | 0.0740 |  |
| 17 | TFDP3 | 0.0010 | 1.39 | 0.0797 |  |
| 18 | OPN1MW2 | 0.0010 | 0 | 0.0840 |  |
| 19 | AVPR2 | 0.0009 | 0.13 | 0.1016 |  |
| 20 | DUSP9 | 0.0009 | 0.60 | 0.1136 |  |
| 21 | MAGEC1 | 0.0009 | 0.86 | 0.1158 |  |
| 22 | IDS | 0.0008 | 0.63 | 0.1386 |  |
| 23 | FHL1 | 0.0008 | 0.25 | 0.1399 |  |
| 24 | MAGEA10 | 0.0008 | 1.58 | 0.1439 |  |
| 25 | F8 | 0.0008 | 0.46 | 0.1440 |  |
