# Supplementary material for: Network Analysis of Differential Expression for the Identification of Disease-Causing Genes
Source: PLoS One. 2009 May 13;4(5):e5526. doi: 10.1371/journal.pone.0005526 (PMC2677677; doi:10.1371/journal.pone.0005526)
Supplement: Table S2 — Top 25 ranked candidate genes in Marfan syndrome (MFS). Marfan syndrome [12] is a heritable connective tissue disorder caused by mutations in the FBN1 gene, and is characterized by increased height, disproportionately long limbs and digits, anterior chest deformity, joint laxity, vertebral column deformity, and other variable skeletal abnormalities, as well as several ocular and cardiovascular features. Candidate genes were chosen from 15q15.3-q22.33 that contains 129 genes including FBN1. These candidate genes were ranked by our new approach, and the top 25 ranked candidate genes are presented here, whereas the top six genes have significant p-values (α = 0.05). FBN1 was ranked on the fifth position with a significant p-value (0.0226). In the ranking we obtained six genes that were significant but not involved in MFS or phenotype related diseases. (0.06 MB DOC) [file pone.0005526.s006.doc]

| **Rank** | **Symbol** | **Score** | **2fold-change** | **p-value** | **Linkage to phenotype** |
| --- | --- | --- | --- | --- | --- |
| 1 | MFAP1 | 0.0152 | 0.15 | 0.0022 |  |
| 2 | CYP19A1 | 0.0139 | 0 | 0.0042 |  |
| 3 | FOXB1 | 0.0111 | 0 | 0.0151 |  |
| 4 | PYGO1 | 0.0105 | 0 | 0.0197 |  |
| **5** | **FBN1** | **0.0103** | 0.22 | **0.0226** | **MFS [12]** |
| 6 | DMXL2 | 0.0087 | 0 | 0.0417 |  |
| 7 | ELL3 | 0.0076 | 0 | 0.0604 |  |
| 8 | SMAD6 | 0.0075 | 0 | 0.0617 |  |
| 9 | RAB8B | 0.0071 | 0 | 0.0718 |  |
| 10 | CGNL1 | 0.0070 | 0 | 0.0725 |  |
| 11 | TMOD3 | 0.0070 | 0 | 0.0731 |  |
| 12 | FGF7 | 0.0067 | 0.14 | 0.0794 |  |
| 13 | LCTL | 0.0067 | 0 | 0.0930 |  |
| 14 | GRINL1A | 0.0065 | 0 | 0.1008 |  |
| 15 | DAPK2 | 0.0065 | 0 | 0.1015 |  |
| 16 | HERC1 | 0.0063 | 0 | 0.1091 |  |
| 17 | PTPLAD1 | 0.0059 | 0.25 | 0.1171 |  |
| 18 | NARG2 | 0.0057 | 0 | 0.1219 |  |
| 19 | MYO5A | 0.0056 | 0.03 | 0.1268 |  |
| 20 | RAB27A | 0.0053 | 0 | 0.1354 |  |
| 21 | ALDH1A2 | 0.0049 | 0 | 0.1505 |  |
| 22 | RAB11A | 0.0048 | 0.10 | 0.1539 |  |
| 23 | MAP1A | 0.0045 | 0 | 0.1678 |  |
| 24 | SPG21 | 0.0044 | 0 | 0.1785 |  |
| 25 | TCF12 | 0.0041 | 0.03 | 0.1939 |  |
