# Supplementary material for: Network Analysis of Differential Expression for the Identification of Disease-Causing Genes
Source: PLoS One. 2009 May 13;4(5):e5526. doi: 10.1371/journal.pone.0005526 (PMC2677677; doi:10.1371/journal.pone.0005526)
Supplement: Table S3 — Top 25 ranked candidate genes in Cystic fibrosis (CF). Cystic fibrosis [13] is an autosomal recessive disorder of epithelial ion transport caused by mutations in the CF transmembrane conductance regulator gene (CFTR), and is characterized by chronic obstructive lung disease, bronchiectasia, and exocrine pancreatic insufficiency. Candidate genes were chosen from chr7q22.1-31.33 that contains 110 genes including CFTR. These candidate genes were ranked by our new approach, and the top 25 ranked candidate genes are presented here, whereas the top nine genes have a significant p-value (α = 0.05). CFTR was ranked in the seventh position with a significant p-value (0.046). In the ranking we obtained seven genes that were significant but not involved in CF or phenotype related diseases. However, out of the top 25 ranked genes we detected four genes that are known to be linked to CF [18]–[21]. (0.06 MB DOC) [file pone.0005526.s007.doc]

| **Rank** | **Symbol** | **Score** | **2fold-change** | **p-value** | **Linkage to phenotype** |
| --- | --- | --- | --- | --- | --- |
| 1 | TFEC | 0.0010 | 1.32 | 0.0073 |  |
| 2 | CAPZA2 | 0.0008 | 0 | 0.0117 |  |
| 3 | WNT2 | 0.0008 | 0 | 0.0118 |  |
| 4 | TAS2R16 | 0.0005 | 0 | 0.0270 |  |
| 5 | MDFIC | 0.0005 | 0.98 | 0.0277 |  |
| 6 | CAV2 | 0.0004 | 0 | 0.0380 |  |
| **7** | **CFTR** | **0.0004** | **0.85** | **0.0460** | **CF[13]** |
| 8 | CTTNBP2 | 0.0003 | 0.82 | 0.0528 |  |
| 9 | LAMB4 | 0.0003 | 0.82 | 0.0536 |  |
| 10 | GNB2 | 0.0003 | 0 | 0.0574 |  |
| 11 | ZNF3 | 0.0003 | 0.81 | 0.0582 |  |
| 12 | SLC13A1 | 0.0002 | 0 | 0.0801 |  |
| 13 | SLC26A5 | 0.0002 | 0 | 0.0802 | [18] |
| 14 | SLC26A4 | 0.0002 | 0 | 0.0803 | [18] |
| 15 | SMURF1 | 0.0002 | 0.73 | 0.0854 |  |
| 16 | ACTL6B | 0.0002 | 0 | 0.1083 |  |
| 17 | MUC17 | 0.0002 | 0.39 | 0.1280 | [19,20] |
| 18 | ZNF277P | 0.0002 | 0 | 0.1330 |  |
| 19 | PUS7 | 0.0002 | 0 | 0.1344 |  |
| 20 | THAP5 | 0.0002 | 0.64 | 0.1349 |  |
| 21 | MUC3B|MUC3A | 0.0001 | 0 | 0.1477 | [21] |
| 22 | DOCK4 | 0.0001 | 0.62 | 0.1541 |  |
| 23 | CADPS2 | 0.0001 | 0 | 0.1546 |  |
| 24 | IMMP2L | 0.0001 | 0 | 0.1561 |  |
| 25 | PIK3CG | 0.0001 | 0 | 0.1652 |  |
