# Supplementary material for: Network Analysis of Differential Expression for the Identification of Disease-Causing Genes
Source: PLoS One. 2009 May 13;4(5):e5526. doi: 10.1371/journal.pone.0005526 (PMC2677677; doi:10.1371/journal.pone.0005526)
Supplement: Table S4 — Top 25 ranked candidate genes in Becker muscular dystrophy (BDM). Becker muscular dystrophy [14] is a X-linked progressive myopathy caused by mutations within the DMD gene, and is characterized by muscle wasting and weakness, and in some cases with mental impairment. Candidate genes were chosen from chrXp22.33-21.1 that contains 116 genes including DMD. These candidate genes were ranked by our new approach, and the top 25 ranked candidate genes are presented here, whereas the top two genes have significant p-values (α = 0.05). DMD ranked on the second position with a significant p-value (0.0272). The other significant gene is not involved in BMD or in a phenotype related disease. (0.06 MB DOC) [file pone.0005526.s008.doc]

| **Rank** | **Symbol** | **Score** | **2fold-change** | **p-value** | **Linkage to phenotype** |
| --- | --- | --- | --- | --- | --- |
| 1 | VCX2 | 0.0771 | 0 | 0.0066 |  |
| **2** | **DMD** | **0.0747** | **0.34** | **0.0272** | **BMD [14]** |
| 3 | GK | 0.0735 | 0 | 0.0537 |  |
| 4 | PRKX | 0.0734 | 0 | 0.0567 |  |
| 5 | PCYT1B | 0.0722 | 0.52 | 0.0978 |  |
| 6 | GPR143 | 0.0721 | 0 | 0.1032 |  |
| 7 | ACE2 | 0.0719 | 0 | 0.1155 |  |
| 8 | ASPGX2 | 0.0718 | 0 | 0.1185 |  |
| 9 | XK | 0.0713 | 0 | 0.1432 |  |
| 10 | VCX | 0.0713 | 0 | 0.1489 |  |
| 11 | PHEX | 0.0709 | 0 | 0.1702 |  |
| 12 | REPS2 | 0.0709 | 0.14 | 0.1719 |  |
| 13 | ASMT | 0.0708 | 0 | 0.1770 |  |
| 14 | MAGEB2 | 0.0708 | 0 | 0.1817 |  |
| 15 | S100G | 0.0706 | 0 | 0.1950 |  |
| 16 | CD99 | 0.0705 | 0 | 0.2051 |  |
| 17 | IL1RAPL1 | 0.0705 | 0.22 | 0.2053 |  |
| 18 | SHOX | 0.0703 | 0 | 0.2177 |  |
| 19 | FAM47A | 0.0702 | 1.09 | 0.2235 |  |
| 20 | GPR64 | 0.0702 | 0 | 0.2236 |  |
| 21 | ZFX | 0.0702 | 0.27 | 0.2267 |  |
| 22 | STS | 0.0702 | 0 | 0.2279 |  |
| 23 | SMPX | 0.0702 | 0 | 0.2280 |  |
| 24 | ARHGAP6 | 0.0701 | 0 | 0.2309 |  |
| 25 | PIR | 0.0701 | 0 | 0.2323 |  |
