# Supplementary material for: Network Analysis of Differential Expression for the Identification of Disease-Causing Genes
Source: PLoS One. 2009 May 13;4(5):e5526. doi: 10.1371/journal.pone.0005526 (PMC2677677; doi:10.1371/journal.pone.0005526)
Supplement: Table S5 — Top 25 ranked candidate genes from chr5q11.2 in Stein-Levental syndrome. Stein-Levental syndrome [22] is a oligogenic hormonal disorder among women putatively related with the FST gene, and is characterized by hyperandrogenism, chronic anovulation and associated with obesity. Candidate genes were chosen from chr5q11.2 that contains 25 genes including the candidate gene FST [24]. These candidate genes were ranked by our new approach, whereas only the top gene has a significant p-value (α = 0.05). FST was ranked in the second position with a p-value of 0.056, and we received only one significant gene (DEAD box 4) that is a plausible candidate gene for Stein-Levental syndrome. (0.06 MB DOC) [file pone.0005526.s009.doc]

| **Rank** | **Symbol** | **Score** | **2fold-change** | **p-value** | **Linkage to phenotype** |
| --- | --- | --- | --- | --- | --- |
| 1 | DDX4 | 0.0524 | 0.54 | 0.0135 |  |
| **2** | **FST** | **0.0424** | **0.36** | **0.0560** | **Stein-Levental syndrome [24]** |
| 3 | ESM1 | 0.0396 | 0.42 | 0.1176 |  |
| 4 | GZMA | 0.0388 | 0.07 | 0.1388 |  |
| 5 | RAB3C | 0.0379 | 0 | 0.1939 |  |
| 6 | ITGA1 | 0.0373 | 0 | 0.2210 |  |
| 7 | ISL1 | 0.0363 | 0.34 | 0.2693 |  |
| 8 | ITGA2 | 0.0356 | 0.20 | 0.3133 |  |
| 9 | IL6ST | 0.0348 | 0.11 | 0.3528 |  |
| 10 | PPAP2A | 0.0338 | 0.07 | 0.4100 |  |
| 11 | HSPB3 | 0.0325 | 0.21 | 0.4814 |  |
| 12 | PLK2 | 0.0324 | 0.45 | 0.4851 |  |
| 13 | GZMK | 0.0317 | 0.14 | 0.5290 |  |
| 14 | IL31RA | 0.0312 | 0 | 0.5538 |  |
| 15 | PDE4D | 0.0308 | 0.63 | 0.5783 |  |
| 16 | GPBP1 | 0.0295 | 0 | 0.6448 |  |
| 17 | MAP3K1 | 0.0293 | 0 | 0.6524 |  |
| 18 | CCNU | 0.0282 | 0 | 0.7075 |  |
| 19 | ARL15 | 0.0264 | 0.45 | 0.7765 |  |
| 20 | SNAG1 | 0.0262 | 0 | 0.7884 |  |
| 21 | DHX29 | 0.0238 | 0.09 | 0.8556 |  |
| 22 | CDC20B | 0.0233 | 0 | 0.8703 |  |
| 23 | MOCS2 | 0.0210 | 0.02 | 0.9142 |  |
| 24 | NDUFS4 | 0.0195 | 0.01 | 0.9366 |  |
| 25 | SKIV2L2 | 0.0169 | 0.04 | 0.9787 |  |
