# Supplementary material for: Network Analysis of Differential Expression for the Identification of Disease-Causing Genes
Source: PLoS One. 2009 May 13;4(5):e5526. doi: 10.1371/journal.pone.0005526 (PMC2677677; doi:10.1371/journal.pone.0005526)
Supplement: Table S6 — Top 25 ranked candidate genes from chr19p13.2 in Stein-Levental syndrome. Stein-Levental syndrome [22] is a oligogenic hormonal disorder among women putatively associated with the FBN3 gene, and is characterized by hyperandrogenism, chronic anovulation and associated with obesity. Candidate genes were chosen from chr19p13.2 that contains 100 genes including the candidate gene FBN3 [26]. These candidate genes were ranked by our new approach, and the top 25 ranked candidate genes are presented here, whereas only the top gene has a significant p-value (α = 0.05). FBN3 was ranked in the fifth position with a p-value of 0.0595. (0.06 MB DOC) [file pone.0005526.s010.doc]

| **Rank** | **Symbol** | **Score** | **2fold-change** | **p-value** | **Linkage to phenotype** |
| --- | --- | --- | --- | --- | --- |
| 1 | OR7G2 | 0.0546 | 0 | 0.0065 |  |
| 2 | EDG8 | 0.0529 | 0 | 0.0129 |  |
| 3 | ZNF414 | 0.0465 | 0 | 0.0249 |  |
| 4 | ADAMTS10 | 0.0442 | 0 | 0.0382 |  |
| **5** | **FBN3** | **0.0423** | **0** | **0.0595** | **Stein-Levental syndrome [26]** |
| 6 | ZNF653 | 0.0413 | 0 | 0.0754 |  |
| 7 | LASS4 | 0.0406 | 0.66 | 0.0894 |  |
| 8 | ACP5 | 0.0406 | 0.15 | 0.0910 |  |
| 9 | EMR4 | 0.0397 | 0 | 0.1141 |  |
| 10 | ZNF177 | 0.0394 | 0.03 | 0.1233 |  |
| 11 | MBD3L1 | 0.0387 | 0 | 0.1464 |  |
| 12 | MUC16 | 0.0385 | 0.31 | 0.1529 |  |
| 13 | EMR1 | 0.0385 | 0.46 | 0.1558 |  |
| 14 | ZNF491 | 0.0383 | 0 | 0.1677 |  |
| 15 | CCL25 | 0.0377 | 0.54 | 0.2056 |  |
| 16 | COL5A3 | 0.0375 | 0.11 | 0.2140 |  |
| 17 | ELAVL3 | 0.0373 | 0.27 | 0.2220 |  |
| 18 | ZNF559 | 0.0371 | 0 | 0.2319 |  |
| 19 | CD320 | 0.037 | 0.50 | 0.2376 |  |
| 20 | STXBP2 | 0.0362 | 0.19 | 0.2778 |  |
| 21 | C19orf45 | 0.0362 | 0 | 0.2796 |  |
| 22 | CLEC4G | 0.0361 | 0 | 0.2838 |  |
| 23 | ZNF44 | 0.0361 | 0.26 | 0.2870 |  |
| 24 | MCOLN1 | 0.036 | 0.11 | 0.2902 |  |
| 25 | TMED1 | 0.0358 | 0.09 | 0.3040 |  |
