# Supplementary material for: Network Analysis of Differential Expression for the Identification of Disease-Causing Genes
Source: PLoS One. 2009 May 13;4(5):e5526. doi: 10.1371/journal.pone.0005526 (PMC2677677; doi:10.1371/journal.pone.0005526)
Supplement: Table S7 — CPU times for computing the Laplacian Exponential Diffusion Kernel by its definition, the CD and the ICD. CPU times (sec) for computing the Laplacian Exponential Diffusion Kernel by its definition (Equation 1), by CD (Equation 2), and by ICD (Equation 3) with a threshold leading to an error of 7%–10%. The computation were run on a dual Opteron 250 with 16 GB RAM. (0.03 MB DOC) [file pone.0005526.s011.doc]

| Number of nodes in the network | CPU time (sec)  Def. (Eq. 1) | CPU time (sec)  CD (Eq. 2) | CPU time (sec)  ICD (Eq. 3) |
| --- | --- | --- | --- |
| 1000 | 32 | 25 | 1 |
| 2000 | 254 | 199 | 8 |
| 5000 | 3637 | 3269 | 98 |
| 10000 | 34717 | 26544 | 633 |
| 16566 (full network) | out of memory | 114953 | 2048 |
