# Supplementary material for: Network Analysis of Differential Expression for the Identification of Disease-Causing Genes
Source: PLoS One. 2009 May 13;4(5):e5526. doi: 10.1371/journal.pone.0005526 (PMC2677677; doi:10.1371/journal.pone.0005526)
Supplement: Table S8 — Rank and Error of resulting Laplacian Exponential Diffusion Kernel computed by its definition, the CD and the ICD. Rank and Error of resulting Laplacian Exponential Diffusion Kernel computed by its definition, (Equation 1), by CD (Equation 2), and by ICD (Equation 3) with a threshold leading to an error of 7%–10%. The computation were run on a dual Opteron 250 with 16 GB RAM. (0.03 MB DOC) [file pone.0005526.s012.doc]

| Number of nodes in the network | Rank of matrix  Def. (Eq. 1) | Rank of matrix  CD (Eq. 2) | Rank of matrix  ICD (Eq. 3) | Error  ICD |
| --- | --- | --- | --- | --- |
| 1000 | 999 | 999 | 167 | 10,09% |
| 2000 | 1999 | 1999 | 337 | 8,92% |
| 5000 | 4999 | 4999 | 773 | 7,94% |
| 10000 | 9999 | 9999 | 1328 | 7,27% |
| 16566 (full network) | 16516 | 16516 | 1829 | 7,28% |
