# Supplementary material for: Network Analysis of Differential Expression for the Identification of Disease-Causing Genes
Source: PLoS One. 2009 May 13;4(5):e5526. doi: 10.1371/journal.pone.0005526 (PMC2677677; doi:10.1371/journal.pone.0005526)
Supplement: Table S9 — Example for determining an appropriate neighborhood size using the example of data set 1 (FXS [11]). The neighborhood size is controlled by a weighting function (w = exp(−β⋅r). Applying the Fisher omnibus meta-analysis (S = ∑−2 ln (p-value)) for each parameter β, new p-values are generated from a Χ∧2 distribution. The parameter β , for which the smallest p-value is observed (here: β = 0.05), leads to the appropriate neighborhood size for FXS (approx. 150 genes). (0.02 MB DOC) [file pone.0005526.s013.doc]

| Parameter β | Approx. size of neighborhood | Fisher’s Omnibus | χ |
| --- | --- | --- | --- |
| 0.5 | 20 | 230.79 | 0.113 |
| 0.1 | 80 | 230.72 | 0.113 |
| 0.05 | 150 | **239.90** | **0.064** |
| 0.01 | 500 | 239.34 | 0.067 |
| 0.005 | 1000 | 237.57 | 0.078 |
| 0.001 | 4000 | 231.16 | 0.129 |
